# Homeostatic plasticity and burst activity are mediated by hyperpolarization-activated cation currents and T-type calcium channels in neuronal cultures

**DOI:** 10.1038/s41598-021-82775-3

**Published:** 2021-02-05

**Authors:** Anikó Rátkai, Krisztián Tárnok, Hajar El Aouad, Brigitta Micska, Katalin Schlett, Attila Szücs

**Affiliations:** Department of Physiology and Neurobiology, Institute of Biology, Eötvös Loránd University, Budapest, Hungary

**Keywords:** Cell biology, Neuroscience

## Abstract

Homeostatic plasticity stabilizes neuronal networks by adjusting the responsiveness of neurons according to their global activity and the intensity of the synaptic inputs. We investigated the homeostatic regulation of hyperpolarization-activated cyclic nucleotide-gated (HCN) and T-type calcium (Ca_V_3) channels in dissociated and organotypic slice cultures. After 48 h blocking of neuronal activity by tetrodotoxin (TTX), our patch-clamp experiments revealed an increase in the depolarizing voltage sag and post-inhibitory rebound mediated by HCN and Ca_V_3 channels, respectively. All HCN subunits (HCN1 to 4) and T-type Ca-channel subunits (Ca_V_3.1, 3.2 and 3.3) were expressed in both control and activity-deprived hippocampal cultures. Elevated expression levels of Ca_V_3.1 mRNA and a selective increase in the expression of TRIP8b exon 4 isoforms, known to regulate HCN channel localization, were also detected in TTX-treated cultured hippocampal neurons. Immunohistochemical staining in TTX-treated organotypic slices verified a more proximal translocation of HCN1 channels in CA1 pyramidal neurons. Computational modeling also implied that HCN and T-type calcium channels have important role in the regulation of synchronized bursting evoked by previous activity-deprivation. Thus, our findings indicate that HCN and T-type Ca-channels contribute to the homeostatic regulation of excitability and integrative properties of hippocampal neurons.

## Introduction

During development and learning, new synapses are formed or eliminated and synaptic strength is increased or decreased in an activity-dependent manner. Classic forms of Hebbian plasticity, such as long-term potentiation (LTP) or depression (LTD), are some of the most investigated basic mechanisms of learning and memory formation, but their effects could easily destabilize the neuronal network without effective negative feedback regulation. As one of such mechanisms, homeostatic plasticity balances neuronal network activity by allowing the neurons to adapt their intrinsic excitability and synaptic responses according to the intensity of the inputs they experience. These homeostatic changes, eventually, will prevent the network from becoming hypo- or hyperactive^1–4^. Recent studies have also demonstrated the importance of homeostatic plasticity in several neurodegenerative diseases and during the onset of neuropathic pain^5–7^.

Synaptic scaling is the most understood form of homeostatic plasticity, which was first observed in dissociated cortical cultures^8^. Long-term inhibition of GABA_A_ receptors (e.g. by bicuculline, picrotoxin or gabazine) or increased membrane depolarization and firing rate evoked by elevated extracellular potassium ion levels have been shown to induce downscaling of postsynaptic glutamate receptors^9^. As a reverse effect, long-term application of tetrodotoxin (TTX) or AMPA-receptor (AMPAR) inhibitors both abolish electrical activity and lead to synaptic upscaling. Prior studies on cultured hippocampal or neocortical neurons^8^ identified postsynaptic AMPARs as targets of homeostatic adaptations during activity-deprivation. Furthermore, it has been shown that this treatment also alters the intrinsic cellular properties of neurons^10,11^.

Indeed, intrinsic biophysical properties of neurons have been found to exhibit a high degree of plasticity. A complex interplay between synaptic inputs and the integrative/biophysical properties of the postsynaptic neuron translates into the firing output that is arguably the most important manifestation of the adaptive changes occurring at multiple levels. Various forms of intrinsic plasticity have been shown to regulate EPSP (excitatory postsynaptic potential) amplification, voltage threshold of spike initiation, and depolarization of the resting membrane potential. These effects can change intrinsic properties through alteration of specific voltage-gated channels in an activity-dependent manner^12–14^. It has been suggested that intrinsic plasticity links Hebbian and homeostatic plasticity, facilitating the formation of neuronal networks that are sufficiently malleable and stable at the same time^12,13^.

Here we report a novel form of homeostatic regulation of low voltage-threshold T-type Ca-channels as well as the hyperpolarization-activated cyclic nucleotide-gated channels, mediating the T- and h-currents, respectively. To induce homeostatic changes in dissociated and organotypic hippocampal cultures, 48 h of TTX was applied to block neuronal firing. After the removal of TTX, robust burst activity as well as elevated voltage sag and post-inhibitory rebound (PIR) were developed which were regulated by HCN and T-type calcium channels, respectively. Quantitative changes of HCN and Ca_V_3 subunits were analyzed by gene expression analysis and western blotting. Plasma membrane localization of the subunits was investigated by surface biotinylation or by detecting TRIP8b (tetratricopeptide repeat-containing Rab8b interacting) protein level, which is an auxiliary subunit of the HCN channels^15^. HCN1 localization was analyzed by immunohistochemistry in organotypic hippocampal slice cultures. In addition, we investigated functional effects of h-current and T-current upregulation in the integrative properties of neurons via computational modeling. Our findings indicate that HCN and T-type Ca-channels actively contribute to the homeostatic regulation of intrinsic excitability and plasticity in pyramidal neurons and have important role in synchronizing network activity.

## Results

### Chronic silencing of electrical activity increases bursting and mEPSC amplitudes

**Figure 1:**
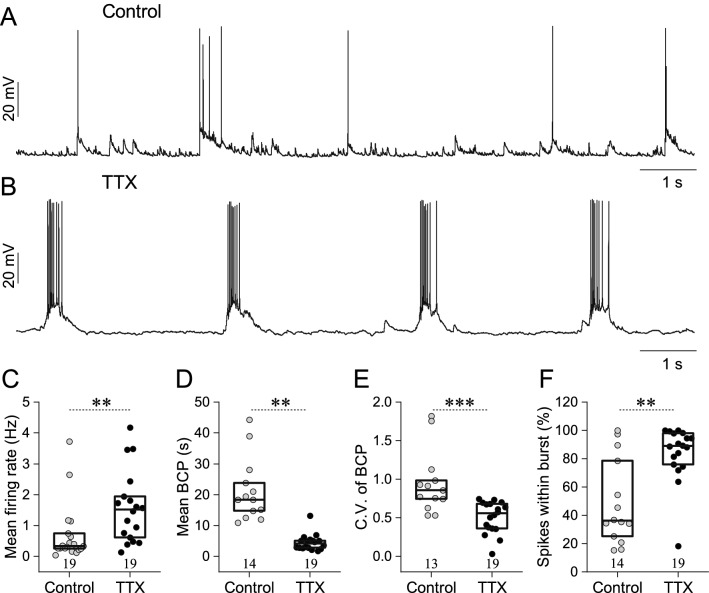
Activity-deprivation by 48 h TTX treatment increases the spontaneous firing rate of cultured hippocampal neurons while regularizing their bursting. Spontaneous membrane potential fluctuations and firing of hippocampal neurons are shown in (**A**) (control) and (**B**) (after 48 h of TTX-treatment). (**C**) Mean firing rate of neurons calculated as the total number of emitted spikes divided by the duration of observation. (**D**) Significant decrease of mean burst cycle period (BCP) is observed in TTX-treated cells relative to the control ones. (**E**) The coefficient of variation (C.V.) of burst cycle periods is also decreased in the TTX group indicating more rhythmic occurrence of bursts. (**F**) The percentage of spikes observed in bursts. Box plots indicate medians and 25–75% range of data. Data are representative of seven (control) and four (TTX) independent cultures. The number of cells analyzed is indicated in the scatter plots (**p < 0.01; ***p < 0.001; Mann–Whitney U test).

As an initial objective, we aimed to characterize the effects of activity-deprivation on the spontaneous firing of dissociated hippocampal neuronal cultures. The prevalent mode of electrical activity in DIV14 to 16 dissociated neuronal circuits is episodic bursting that reflects synchronous activation of a high number of synaptically coupled neurons. Typically, in control cultures, a high percentage of spikes is emitted during episodes of potent excitatory synaptic inputs (compound EPSPs) that can trigger brief bursts in the postsynaptic cell (Fig. 1A), but regular, near periodic activity is generally not observed. On the other hand, more regular, stereotypic bursts developed when cells were previously treated with 1 µM TTX for 48 h (Fig. 1B). Chronic activity-deprivation of hippocampal neurons significantly increased their mean firing rate (Fig. 1C) following the washout of TTX. Additionally, burst cycle period (BCP) values were significantly decreased following activity-deprivation (Fig. 1D). Bursts were also emitted in a more regular pattern as the coefficient of variation of burst cycle periods was significantly reduced relative to the control population (Fig. 1E). Additionally, the percentage of spikes emitted within bursts (thus, the intraburst spike ratio) increased dramatically that indicates the tight synchronization of activity in the network of cultured hippocampal neurons (Fig. 1F).

**Figure 2:**
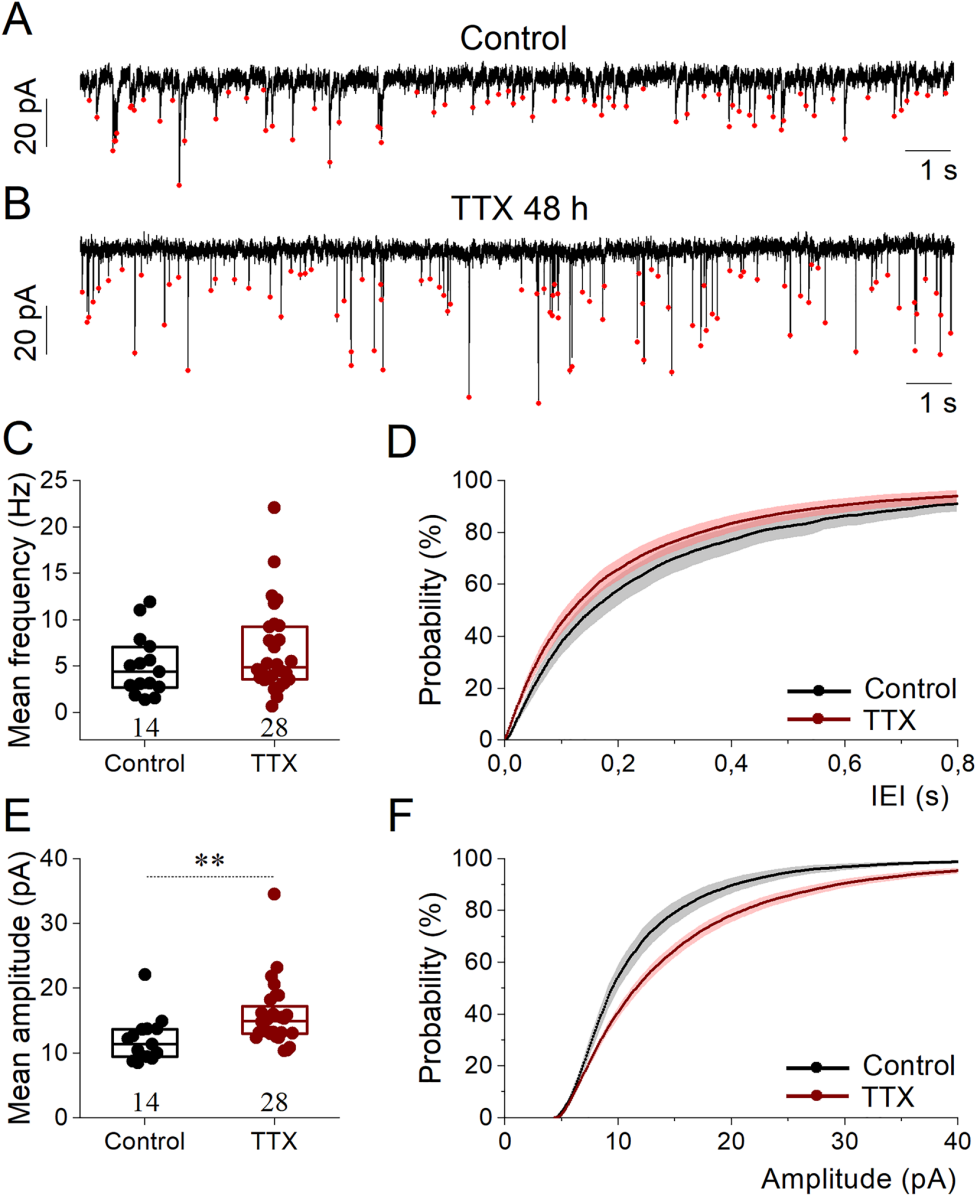
The amplitude of spontaneous excitatory postsynaptic currents is increased by 48 h silencing of cultured hippocampal neurons. Representative voltage clamp recordings of miniature excitatory postsynaptic currents (mEPSCs) of 20 s duration are shown in (**A,B**) from control vs. TTX-treated neurons, respectively. Mean mEPSC frequency data from control and TTX-treated neurons are compared in (**C**). Pooled cumulative probability distributions of inter-event intervals are shown in (**D**). Mean mEPSC amplitudes are compared in (**E**). Cumulative amplitude probability histograms for the two conditions are shown in (**F**). Data are representative of two (control) and four (TTX) independent cultures. Box plots indicate medians and 25–75% range of data. Number of recorded cells is indicated on the graphs. Values are expressed as mean ± SEM (**p < 0.01; Mann–Whitney U test).

While our main focus was on the homeostatic alterations of intrinsic biophysical properties of neurons, we performed additional voltage clamp experiments to verify the known synaptic effects of long-term activity-deprivation in our mouse hippocampal cultures^8^. Miniature postsynaptic currents were recorded at − 60 mV holding potential with 500 nM TTX in the bath to prevent spike mediated neurotransmission. Most neurons in such conditions exhibited fast excitatory current transients reflecting AMPA-receptor activation via spontaneous release (Fig. 2A,B). In agreement with previously published data^8^, the frequency of spontaneous activity was similar in control and TTX-treated cells (Fig. 2C). Accordingly, the cumulative average interevent interval (IEI) probability distributions were similar (Fig. 2D). However, the average amplitude of minis was significantly increased (Fig. 2E), leading to the right shift of the cumulative probability distributions of mini amplitudes (Fig. 2F) after TTX-treatment. These data indicate that average transmitter release probability remained unaffected in TTX-treated cultures while a general upscaling of AMPA-receptor mediated postsynaptic currents was induced. These findings are in good agreement with earlier observations on rat cortical pyramidal neurons^8^ and demonstrate homeostatic regulation of synaptic properties evoked by chronic TTX-treatment.

### Activity-deprivation induces adaptive changes in the intrinsic excitability and physiological properties of cultured hippocampal neurons

**Figure 3:**
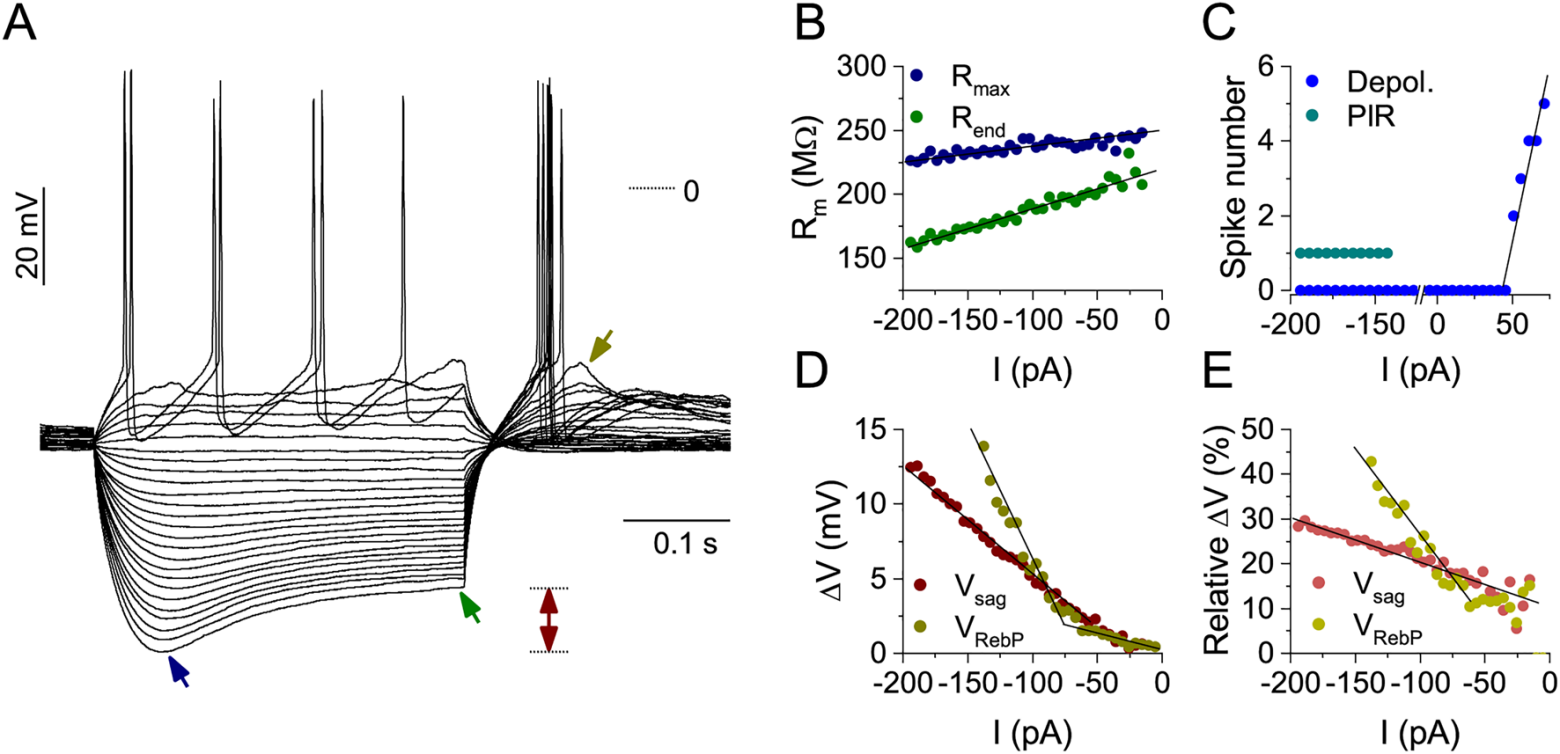
Analysis of the voltage responses of cultured hippocampal neurons under current step stimulation. The neuron exhibits voltage sag and post-inhibitory rebound firing under the injection of negative current steps (**A**). The maximal voltage deflection (blue arrow, ∆V_max_) and the voltage at the end of the current pulse (green arrow, ∆V_end_) are used to obtain the input resistance curves on (**B**) The spike number as a function of the input current is shown in (**C**) (I–O relationship; blue symbols for spikes under positive current steps, teal symbols for PIR spikes). Here the rheobase and the initial slope of the I–O curve (gain) are determined. Voltage sag (maroon) and rebound potential (RebP; dark yellow) as functions of the input current are shown in (**D**) RebP in this cell grows steeply when current pulses more negative than − 100 pA are injected. (**E**) shows the relative voltage sag and relative RebP from the same data. These are calculated by dividing voltage sag and RebP values by their corresponding maximal voltage deflection values (∆V_max_).

**Figure 4:**
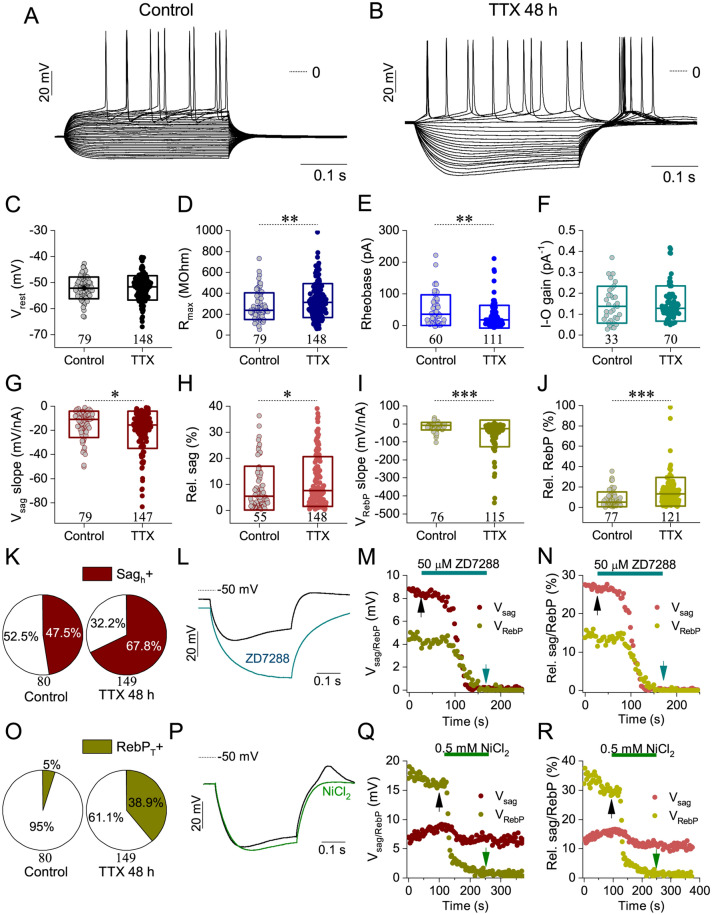
Chronic silencing of electrical activity of hippocampal neurons by 48 h TTX treatment increases their excitability, the degree of their post-inhibitory firing and the magnitude of their voltage sag. Sample voltage traces under current step stimulation from a control and an activity-deprived cell are shown in (**A**,**B**), respectively. (**C**–**J**) Scatter plots display physiological parameters calculated from voltage responses of control and TTX-treated neurons, such as average resting membrane potential (**C**), average input resistance (**D**), rheobase (**E**) and I–O relationship (**F**). The initial slope of the voltage sag vs. current and RebP versus current relationship (**G**,**I**, respectively) and the relative voltage sag (**H**) and the relative RebP (**J**) are also presented. The box plots next to the data points indicate median and 25-75% range of data. Pie charts in (**K**,**O**) illustrate the percentage of cells that are visually classified as exhibiting voltage sag (**K**) or T-current mediated PIR (**O**), respectively. (**L**) Voltage responses of a TTX-treated neuron exhibiting clear voltage sag in normal ACSF (black trace) and monoexponential voltage deflection (teal) after application of HCN channel blocker ZD7288. (**M**,**N**) show the absolute (**M**) and relative (**N**) voltage sag and rebound potential as the function of time while stimulating the neuron in 3 s intervals using a fixed − 150 pA current step. Both parameters drop quickly after the introduction of ZD7288 at t = 70 s. (**P**) Bath application of NiCl_2_ (green) removes the post-inhibitory T-current mediated rebound depolarization while voltage sag is only slightly influenced by NiCl_2_. (**Q**,**R**) display the time courses of the voltage sag and rebound potential parameters during the NiCl_2_ experiment, respectively. Data are derived from 13–13 independent cultures. Number of recorded cells is indicated on the graphs (*p < 0.05; **p < 0.01; ***p < 0.001; (**C**) Independent samples t-test, (**D**–**J**) Mann–Whitney U test).

To evaluate potential homeostatic changes in the intrinsic properties of cultured hippocampal neurons upon chronic activity-deprivation, we calculated multiple passive and active physiological parameters from their voltage responses under incrementing levels of the injected current. Typically, membrane resistance curves were obtained by sampling the voltage responses of neurons at two points: at the location of maximal voltage deflection and 5 ms before the end of the stimulus current step (Fig. 3A). Therefore, two sets of resistance data were obtained resulting in two trajectories (Fig. 3B) with varying degree of separation depending on the magnitude of voltage sag. Intrinsic excitability of the neurons was characterized by input–output relationship plots that show the number of emitted spikes as a function of the stimulus current (Fig. 3C). Absolute and relative voltage sag as well as rebound potential values were also sampled at various levels of stimulus current and linear fitting was utilized to characterize the dependence of such parameters upon the input current (Fig. 3D,E, respectively). The relative voltage sag/rebound potential parameters are calculated by dividing the absolute voltage sag/rebound potential values by the corresponding maximal voltage deflections. Representative voltage traces of hippocampal neurons cultured either in normal medium or after 48 h of TTX-evoked activity-deprivation are shown in Fig. 4A,B, respectively. Within our control cultures, most of the cells were classified as regular firing types (Fig. 4A). In contrast, following 48 h of TTX-treatment, an increased number of neurons displayed voltage sag, rebound depolarization and even post-inhibitory firing (Fig. 4B,K,O). These recordings indicate that voltage-gated membrane currents that are activated at hyperpolarized membrane potentials are upregulated upon long-term activity-deprivation of the cells.

Multiple physiological parameters of hippocampal neurons from control and activity deprived cultures were additionally investigated (Fig. 4C–J). Resting membrane potential data followed normal distribution and did not differ significantly between the two groups (Fig. 4C). However, when comparing the voltage sag exhibiting and -lacking neurons within each group, TTX-treatment resulted in significantly depolarized resting membrane potential in sag + cells (Supplementary Fig. S1A). The input resistance of the activity deprived neurons, in average, increased (Fig. 4D), which is often associated with elevated excitability. At the same time, the rheobase (current threshold for spike emission) was significantly lowered in TTX-cells relative to the controls (Fig. 4E) but no changes in the average slope of I–O curves (I–O gain) were detected (Fig. 4F).

As indicated above, linear fits of the voltage sag vs. current plots yielded parameters that characterized the magnitude of voltage sag, thus the strength of the hyperpolarization-activated cation currents. The average slope of the regression line fitted to the absolute voltage sag was more negative for TTX-treated cells than for controls (Fig. 4G). In addition, the relative voltage sag was also increased in TTX-treated cells (Fig. 4H).

We found that rebound potential (RebP) vs. current data can be well fitted by double linear functions (see Fig. 3D,E). Figure 4I,J show slope parameters obtained from fitting absolute and relative RebP (thus, RebP values normalized with membrane resistance) values below − 60 pA current levels. Both parameters were significantly changed upon activity-deprivation. In summary, our observations verified that 2 days of activity-deprivation induced a compensatory increase in intrinsic excitability in dissociated hippocampal neurons.

### Voltage sag and rebound potential are mediated by HCN and T-type calcium channels

Voltage sag is commonly associated with the action of HCN channels mediating the h-current^16^. To test the impact of h-current in shaping the behavior of hippocampal neurons, we applied 50 μM ZD7288 to pharmacologically block HCN channels and immediately started monitoring the neurons’ voltage responses under constant current step stimulation (Fig. 4L–N, Supplementary Fig. S1B–D). Inhibition of the h-current removed the voltage sag initially present in the response and slightly shifted the resting membrane potential to more negative levels (Fig. 4L). It is notable that both the voltage sag and RebP are quickly and reliably eliminated by blocking the h-current (Fig. 4M,N). Importantly, acute application of 50 μM ZD7288 did not change the resting membrane potential of voltage sag-negative cells (Supplementary Fig. S1B–D) that further strengthens the link between voltage sag, rebound depolarization and the presence of functional HCN channels.

Post-inhibitory rebound (PIR) or firing requires the action of T-type calcium current. Under T-current mediated rebound firing, spikes appear on top of a characteristic bump that is more pronounced than the h-current mediated RebP alone. This behavior has been described for multiple neuronal phenotypes that express low-threshold Ca-currents (I_T_) via activation of Ca_V_3 channels^17^. In order to clarify the ZD7288-dependent possible effects on the T-current, we measured from the same neurons T-current activation with voltage clamp and the post-inhibitory rebound appearance using a current step protocol in the presence of 50 μM extracellular ZD7288 blocker (see Supplementary Fig. S2). Our results convincingly show that ZD7288 does not block the T-current.

To clarify the involvement of T-current in promoting the post-inhibitory firing and RebP-boosting effect, we used 0.5 mM NiCl_2_ as a potent blocker of voltage-gated T-type calcium channels under current step stimulation (Fig. 4P–R). NiCl_2_ rapidly eliminated the post-inhibitory bump while leaving much of the negative current response and voltage sag intact (see the sample trace on Fig. 4P as well as temporal changes upon NiCl_2_ perfusion on Fig. 4Q,R). These observations clearly show the involvement of T-current in shaping and amplifying the rebound potential.

### Expression of HCN and T-type Ca-channels in dissociated hippocampal neuronal cultures

**Figure 5:**
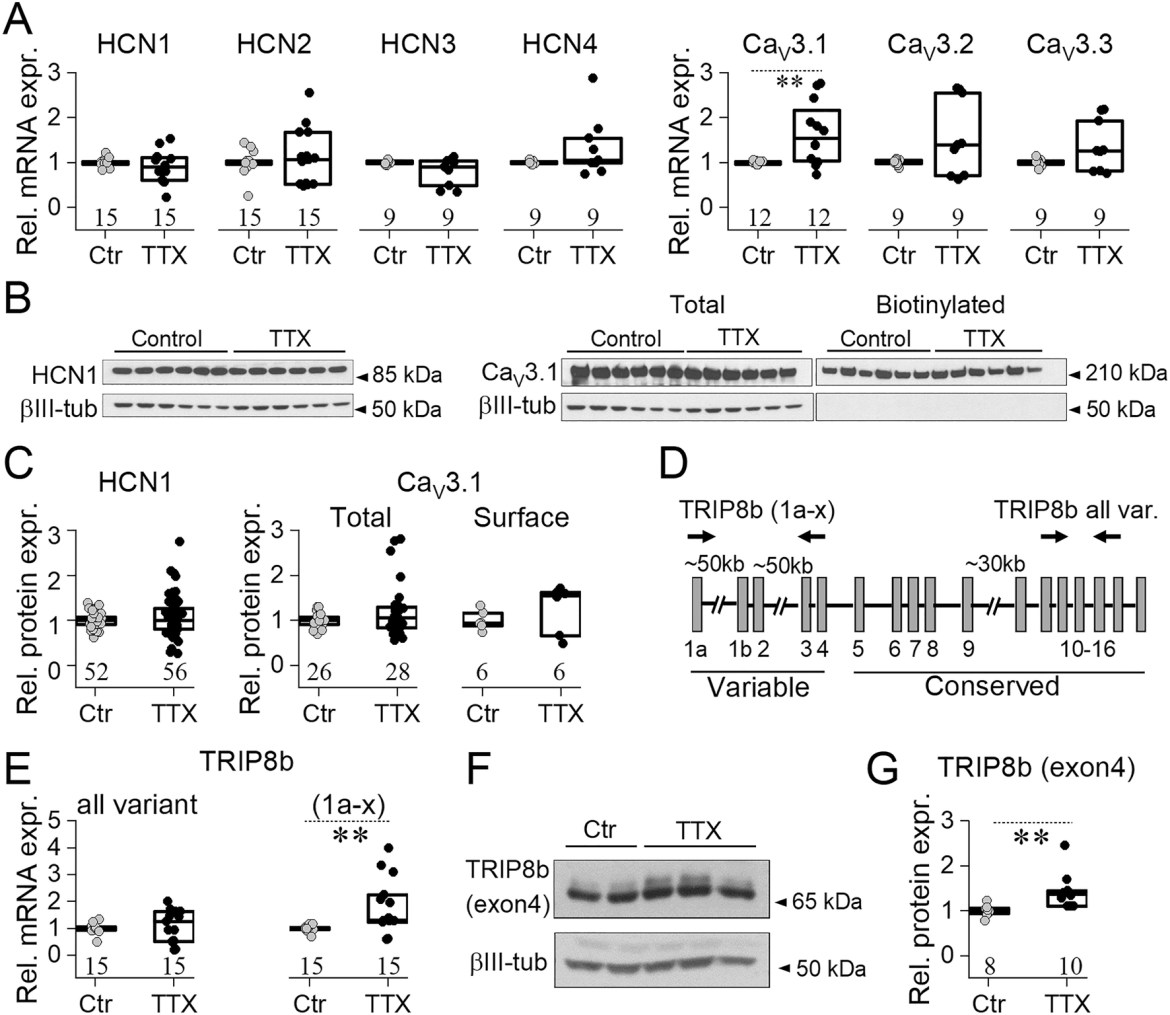
Homeostatic upscaling regulates HCN1 via TRIP8b. (**A**) Analyzing the relative mRNA expression of HCN and Ca_V_3 subunits revealed that only Ca_V_3.1 mRNA level is increased upon chronic TTX treatment. (**B**) Representative western blots show HCN1 and Ca_V_3.1 protein signals in whole cell (total) or in biotinylated cell lysates from control and TTX-treated hippocampal neuronal cultures. (**C**) Quantification of HCN1 and Ca_V_3.1 western blot data, displaying normalized HCN1 and total Cav3.1 levels to their corresponding βIII-tubulin levels or precipitated (surface) Cav3.1 levels, normalized to the average of control sample intensities developed within the same blot. (**D**) Schematic illustration of the TRIP8b gene. Arrows indicate localization of the used primers in RT-qPCR. (**E**) Quantification of TRIP8b mRNA expression shows a significant increase in the TRIP8b (1a-x) splice variant. (**F**) Representative western blots from control and TTX-treated culture lysates to detect TRIP8b (exon4) level. (**G**) Deprivation of spontaneous firing activity significantly increased the TRIP8b (exon4) protein level. In case of RT-qPCR, relative expression levels were normalized to GAPDH while βIII-tubulin was used as loading control in western blots. Box plots indicate medians and 25–75% range of data. RT-qPCR data are derived from 4 (HCN1, HCN2, TRIP8b all variant, TRIP8b (1a-x)) and 3 (HCN3, HCN4, Ca_V_3.1, Ca_V_3.2, Ca_V_3.3) independent cultures while protein levels were detected in cell lysates obtained from 19 (HCN1), 11 (total Ca_V_3.1), 2 (biotinylated Ca_V_3.1) and 4 (TRIP8b (exon4)) independent cultures. Number of recorded cells is indicated on the graphs (**p < 0.01). For statistical analyses, independent samples t-tests (HCN1, HCN2, Ca_V_3.1, Ca_V_3.2, Ca_V_3.3 mRNA levels on (**A**)) or Mann–Whitney U tests (HCN2, HCN4 mRNA levels on (**A**,**C**,**E**,**F**)) were used.

Based on our electrophysiological observations, we investigated the expression of HCN and T-type Ca channels in control and TTX-treated dissociated hippocampal neuronal cultures. The obtained RT-qPCR data show that among all four HCN subunits expressed, HCN1 and HCN2 subunits were the most abundant forms in our cell cultures (∆Cq(HCN1) = 4.50, ∆Cq(HCN2) = 4.74, ∆Cq(HCN3) = 7.87 and ∆Cq(HCN4) = 7.84). Among the T-type calcium channel subunits, Ca_V_3.1 and Ca_V_3.2 channel expression levels were the highest (∆Cq(Ca_V_3.1) = 7.74, ∆Cq(Ca_V_3.2) = 7.28 and ∆Cq(Ca_V_3.3) = 8.07). Interestingly, activity-deprivation significantly increased Ca_V_3.1 mRNA expression but did not alter the mRNA level of the other T-type calcium channel subunits or any HCN channels (see Fig. 5A).

In order to test how deprivation of neuronal activity affects HCN and Ca_V_3 protein levels, western blot experiments were carried out for the most abundant subtypes. Unfortunately, available antibodies for Ca_V_3.2 failed to detect specific bands in our hands (data not shown). On the other hand, Ca_V_3.1 and HCN1 protein levels were readily detected in whole cell lysates (Fig. 5B,C, Supplementary Fig. S3). Total HCN1 or Ca_V_3.1 signal intensity values were normalized to their corresponding βIII-tubulin levels. All ratios detected within the same blots were normalized to the average of the control values. According to our results, relative total protein levels were not changed upon long-term TTX treatment (Fig. 5B,C, Supplementary Fig. S3). Additionally, surface biotinylation was carried out to selectively label and precipitate Ca_V_3.1 subunits from the plasma membrane. Biotinylated Ca_V_3.1 signal intensity values were normalized to the corresponding total Ca_V_3.1 signal from the same sample. All ratios detected within the same blots were normalized to the average of the control values and displayed as “Surface” on Fig. 5C. Our data did not reveal significant differences between surface-localized Ca_V_3.1 signal in control or TTX-treated cell lysates (Fig. 5B,C, Supplementary Fig. S3).

It is known that TRIP8b (tetratricopeptide repeat-containing Rab8b interacting protein, also known as Peroxisomal Biogenesis Factor 5 Like, PEX5L) is a brain-specific auxiliary subunit of the HCN channels with more than 10 splice variants^15,18^. Out of these, splice variants containing 1a, 1a-4, 1b-2 or 1b-2-4 exons are the most abundant in nervous tissue^15^. Among these, TRIP8b splice isoforms containing exons 1a and 4, designated as TRIP8b (1a-4), have specific importance in regulating HCN1 trafficking and plasma membrane localization^15,19,20^. Primers binding within the conserved C-terminal domain of TRIP8b mRNA (indicated as “all variants”, Fig. 5D) did not detect a change in TRIP8b expression between control and TTX-treated cultures. On the other hand, primers detecting the splice variants containing the 1a domain within the variable region of TRIP8b (referred as 1a-x, Fig. 5D), TRIP8b (1a-x) revealed significantly increased mRNA levels upon neuronal silencing (Fig. 5E).

Using a TRIP8b exon 4-specific antibody, we were able to show that exon 4 containing TRIP8b protein level was also elevated upon long-term TTX treatment (Fig. 5F,G, Supplementary Fig. S3). Exon 4 is also found in the TRIP8b (1b-2–4) splice variant, which has been reported to decrease HCN1 surface localization^15^. On the other hand, it is important to note that the expression of TRIP8b 1b splice variant was not detected using splice variant specific antibody (data not shown). All these findings indicate that chronic blockade of neuronal activity in hippocampal cultures enhanced the expression of TRIP8b (1a-4) splice isoform, which is known to regulate plasma membrane localization of HCN1 subunits^19^.

### HCN1 spatial redistribution is induced by activity-deprivation in organotypic hippocampal slice cultures

Proximal translocation of HCN1 channels along the dendrites has been reported to increase excitability^21^. As dissociated cell cultures do not preserve the original connections within the brain, we decided to use organotypic hippocampal slice cultures to investigate how 48 h of 1 μM TTX treatment affected CA1 pyramidal neurons.

**Figure 6:**
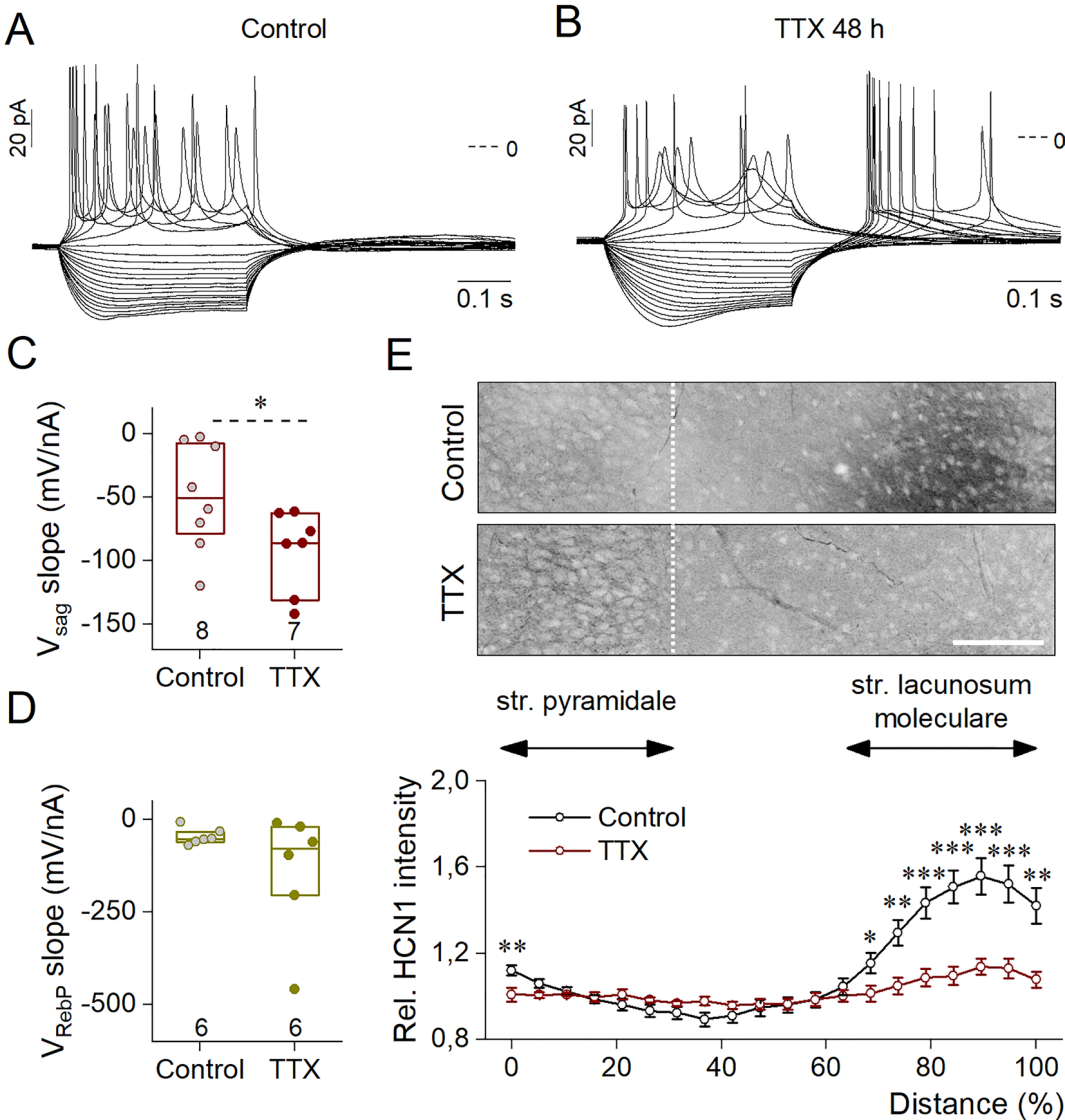
Representative voltage traces obtained from organotypic slice cultures under current step stimulation from a control (**A**) and an activity-deprived (**B**), CA1 pyramidal neuron at DIV17 and DIV14, respectively. The voltage sag slope parameter (**C**) became significantly more negative, while the slope of RebP (**D**) changed to a less degree following TTX treatment. Number of recorded cells is indicated on the graphs, where box plots indicate medians and 25–75% range of data. (**E**) Activity deprivation induced a more proximal redistribution of HCN1 from the str. lacunosum moleculare (str. l.m.). Representative images were processed identically and display inverted a-HCN1 immunofluorescence from control and TTX-treated slices. Dashed lines indicate the edge of str. pyramidale (str. pyr.). Scale bar represents 100 μm. At the bottom, graph indicates relative HCN1 signal intensity values along 100 μm wide lines oriented perpendicular between str. pyr. and str. l.m., normalized to the average HCN1 intensity within the str. pyr. of the same slice. Data were calculated form 16 (control) or 11 (TTX-treated) intensity profiles and are expressed as mean ± SEM. Altogether, 6 (**C**,**D**) or 4 (**E**) independent organotypic hippocampal slice cultures were used for the measurements. Statistical analysis was performed with Mann–Whitney U test (**C**,**D**) or independent samples t-test ((**E**); *p < 0.05; **p < 0.01; ***p < 0.001).

We measured the voltage responses of CA1 neurons using current steps from control and TTX-treated DIV12–16 slice cultures (Fig. 6A–D). In all cases, recorded neurons were filled with biocytin to reveal their localization (data not shown). Only those recordings were selected for the analyses where the pyramidal morphology and CA1 localization were verified. As indicated by the sample traces, TTX-treated CA1 neurons exhibited pronounced voltage sag and post-inhibitory firing (Fig. 6A,B). Similarly to data obtained from dissociated cultures (see Fig. 4G), strength of the voltage sag was significantly increased upon activity-deprivation (Fig. 6C). The slope of the rebound potential did not change significantly (Fig. 6D) which can be due to post-inhibitory spikes evident during nearly all measurements, hindering the fitting of the RebP values. However, the increased propensity of PIR firing was consistent with the upregulation of T-type Ca-current in the TTX-treated preparations. Taken together, our patch clamp results proved that activity-deprivation induced similar changes in the intrinsic properties of neurons developing in dissociated cultures or in organotypic hippocampal slices.

Our western blot results suggested a possible redistribution of HCN1 channels in activity-deprived neurons. To test this, we performed HCN1 immunohistochemistry in DIV14-16 organotypic slice cultures (Fig. 6E). As earlier reported^20,22^, the strongest HCN1 signal was observed in the str. lacunosum moleculare of the CA1 region of the control slices. In case of TTX-treated slices, on the other hand, distal dendrites were less stained while in the str. pyramidale, a stronger signal was detected in comparison to the control (see Fig. 6E, with both images taken and processed uniformly). To quantify these differences, intensity profile analysis was applied by measuring average HCN1 intensity along a 100 μm wide line fitted perpendicularly in the CA1 region from the str. pyramidale to str. lacunosum moleculare in identically captured control and TTX-treated images. Local intensity values along the line were determined in every 5% of the total length and were normalized to the average intensity values determined within the str. pyramidale of the same slice. In case of control slices, relative HCN1 intensity was significantly elevated within the distal region of str. lacunosum moleculare while signal intensity distribution showed an even profile in activity-deprived organotypic cultures.

These findings clearly prove that 48 h of TTX treatment induced spatial redistribution of HCN1 channels and increased excitability in CA1 pyramidal neurons.

### HCN and T-type calcium channels have an important role in neuronal bursting induced by long-term deprivation of activity-dependent neurotransmission

**Figure 7:**
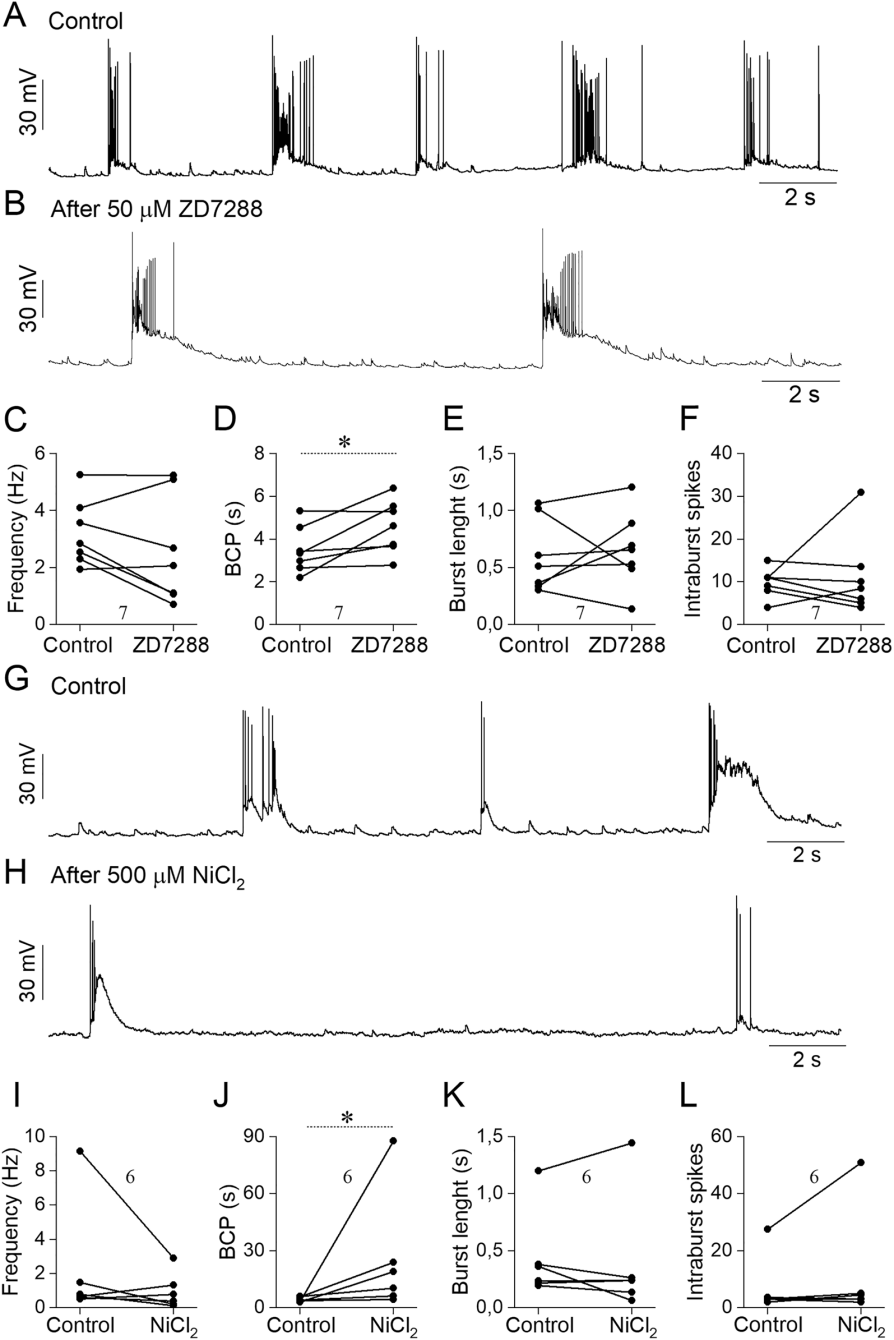
HCN and T-type calcium channels are involved in the increased bursting activity during homeostatic upscaling. Spontaneous firing activity was registered from the same TTX-treated neuron before (**A**) and after (**B**) the HCN channel blocking (50 μM ZD7288). Blocking of the HCN channels does not affect firing frequency (**C**), burst length (**E**) or the number of intraburst spikes (**F**), but significantly increases the burst cycle period **(D**). Representative figures (**G**,**H**) show the T-type calcium channel-dependent effect on the firing activity during TTX treatment. Blocking of the T-type calcium channels by 500 μM NiCl_2_ significantly decreased the bursting activity (**J**), but did not change the firing frequency (**I**), the burst length (**K**) or the number of intraburst spikes (**L**). Data are representative of three independent cultures. The number of cells analyzed is indicated above or below data in the graphs. Statistical analysis was performed with paired sample t-test (*p < 0.05).

Next, we wished to investigate the functional role of HCN (Fig. 7A–F) and T-type calcium (Fig. 7G–L) channels in regulating the spontaneous network activity of hippocampal neurons. Application of 50 µM ZD7288 had only a minor effect on the average firing frequency (Fig. 7C) and did not affect the length of bursts (Fig. 7E) or the number of intraburst spikes (Fig. 7F), but significantly increased the burst cycle period (Fig. 7D). These observations show that pharmacological inhibition of HCN channels lowers overall activity by reducing the frequency of burst episodes. Blocking of the T-type calcium channels evoked a more robust change in the bursting activity of the analyzed cells (Fig. 7G,H). While application of 0.5 mM NiCl_2_ did not significantly change the average firing rate (Fig. 7I), the length of the developing bursts (Fig. 7K) or the number of spikes in burst (Fig. 7L), it significantly increased the burst cycle period (Fig. 7J). Notably, 50 µM ZD7288 did not disrupt the excitatory synaptic transmission, as temporal and amplitude distributions of mEPSCs were similar both in control and ZD7288-treated cells (see in Supplementary Fig. S1E).

Taken together, our data indicate that both HCN and T-type calcium channels have a potent role in regulating the generation of bursts.

### Computational modeling confirms the importance of HCN and T-type Ca-channels in homeostatic adaptations

**Figure 8:**
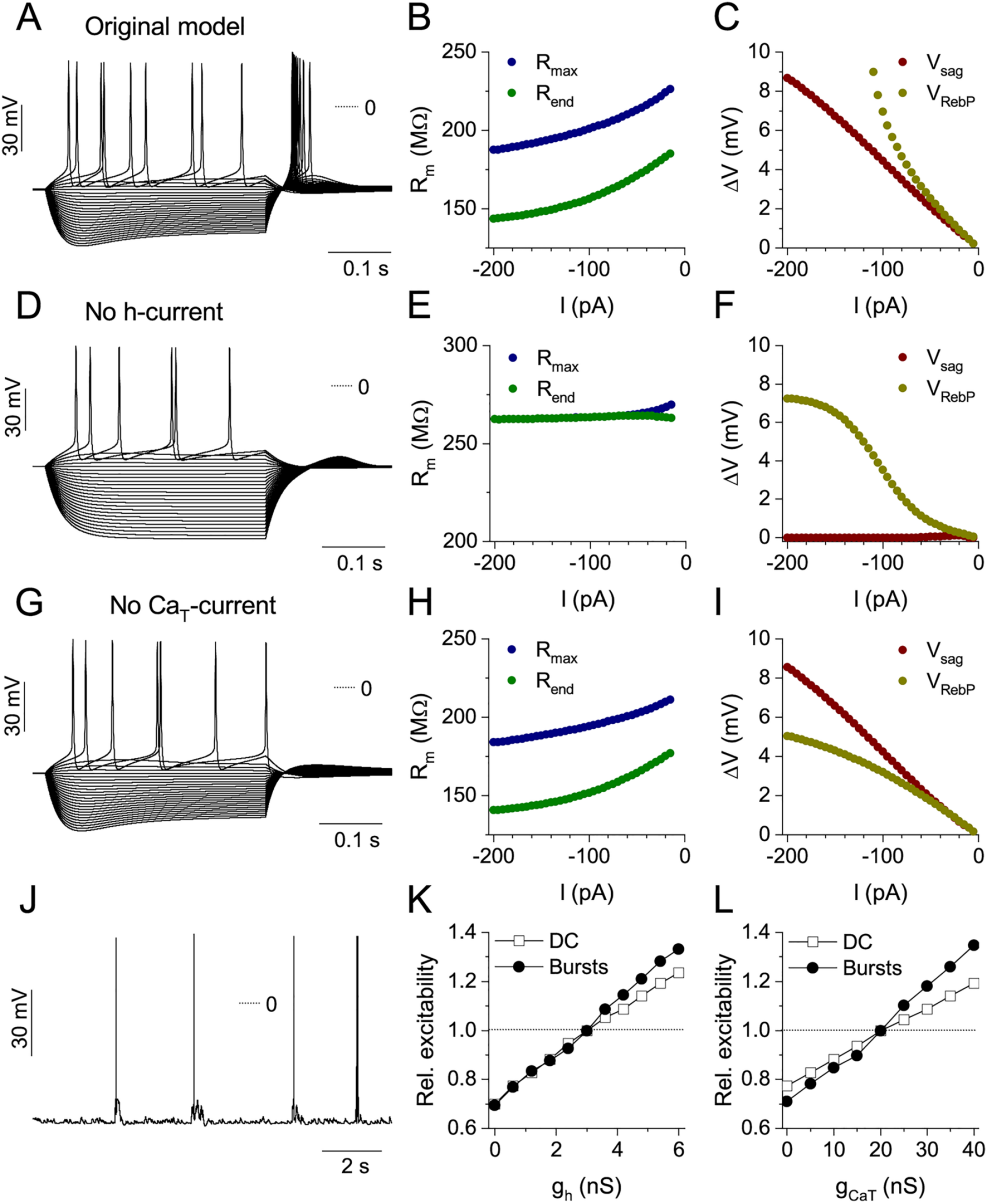
The computational model reproduces the physiological effects of h- and T-currents and the homeostatic changes observed under activity-deprivation. (**A**) shows the voltage responses of the generic model neuron expressing moderate levels of intrinsic h- and T-currents while (**D**,**G**) show the responses after selectively removing the h- or T-current, respectively. Panels (**B**,**E**,**H**) demonstrate the membrane resistance plotted against the injected current for the three conditions while (**C**,**F**,**I**) display voltage sag and rebound potential relationships from the same data. RebP increases steeply at current levels more negative than − 60 pA in the generic model (**C**). Moderate RebP is still observed when the T-current alone is removed (**I**). Firing output of the model is shown when network bursting type synaptic input is used (**J**). Relative cumulative spike numbers are plotted against the maximal conductance of the h- and T-currents ((**K**,**L**), see text).

The role of various voltage-dependent currents in regulating the intrinsic excitability of neurons has been a topic of electrophysiological studies and computational modeling^23–25^. The h-current, in particular, is interesting because both positive and negative impacts have been reported^10^. Computational modeling of single neurons can assist to elaborate on this idea as well as to predict their firing responses under simulated synaptic inputs such as the ones occurring during synchronous network activation in hippocampal cultures. The generic CA1 neuron model (Fig. 8A) in our simulations consisted of one axonal, one somatic and one proximal dendritic compartment and a total of 7 voltage-gated membrane currents were distributed across the compartments. In addition to the standard spike generating currents, we included the non-inactivating M-type K-current, the T-, h- and persistent Na-current as well as one type of Ca-dependent K-current.

We aimed to reconstruct the physiological effects of homeostatic adaptations observed in biological neurons by selectively manipulating the maximal conductances of the h- and T-current in the model and performing simulated current step experiments (Fig. 8). The voltage responses of the generic model are shown in Fig. 8A. This neuron has moderate levels of both the h- and T-current and the characteristic voltage sag and post-inhibitory firing are both reproduced. In this respect, the model is more representative of the activity-deprived neurons that we routinely patched in our TTX-treated cultures. The model also reproduces the general behavior of the membrane resistance in relation to the stimulus current (Fig. 8B, separated curves) and the steeply rising RebP curve below − 60 pA current levels (Fig. 8C).

Removal of the h-current from the model mimics the effects of ZD7288 (Fig. 8D–F) as we demonstrated in the biological neurons (see Fig. 4L–N). In particular, the depolarizing voltage sag disappears and the resting membrane potential shifts to a slightly more hyperpolarized level (from − 54.4 to − 57.5 mV; Fig. 8E). Nevertheless, rebound potential mediated here solely by the T-current, remains in the model response (Fig. 8F), although PIR spikes are not emitted in such conditions. Clearly, the activation of the T-current alone is not sufficient to bring the membrane potential to spike threshold, and the contribution of the h-current is also necessary for the PIR firing behavior.

Simulation of the effect of NiCl_2_ (see in Fig. 4P–R) can be performed by simply removing the T-current from the model as shown in the voltage responses in Fig. 8G. Figures 8B,H demonstrating the membrane resistance remain very similar under such manipulation. The voltage sag vs. current function is also unaffected by T-current removal, but the rebound potential curve is now far less steep than that of the generic model (Fig. 8C vs. I). This is, again, in good agreement with our findings on the biological neurons.

We also note that the impact of T-current in regulating the rebound depolarization becomes stronger when more negative current steps are applied (Fig. 8F). In contrast, the effect of the h-current is more uniform, the slope of the RebP vs. current fit does not change dramatically in the − 200 to 0 pA range (Fig. 8I). Therefore, the slope of the linear fit applied to points below − 60 pA is very informative in separating the effects of the h- vs. T-current and we can use this analysis to detect neurons with upregulated T- or h-currents.

Next, we investigated the h- and T-current dependent changes in intrinsic excitability in two scenarios: either using the standard current step stimulation (static I-O curve; indicated as “DC” in Fig. 8K,L) or subjecting the model neurons to simulated synaptic bombardment (network bursts; indicated as “Bursts” in Fig. 8K,L) that represent conditions hippocampal neurons experience in culture. In the latter case, we simulated synchronized synaptic inputs which are common in the previously silenced hippocampal cultures and calculated the model neurons’ voltage responses (Fig. 8J). Firing rate of the neurons under synaptic inputs was surveyed by gradually incrementing the synaptic conductance of the AMPA- and GABA-inputs and counting the spikes emitted by the neuron in the subsequent trials (dynamic I–O curves). Such simulations were performed for various conductance levels of the intrinsic h- and T-currents and the excitability profiles shown in Fig. 8K,L were obtained. Normalized cumulative spike numbers were calculated in the following way: first we counted the total number of emitted spikes during the stimulus protocol (either the standard current steps or the synaptic bombardment used); next we divided the spike counts at different levels of the intrinsic h- or T-current by the spike count at the original level. Such normalized spike counts also referred to as relative excitability (Fig. 8K,L) increased linearly as the maximal conductances of the two voltage-gated currents were upregulated. As this analysis reveals, upregulation of these currents has a positive effect on firing responses, but the number of spikes increases more steeply when the neurons experience synaptic inputs than when subjected to current steps (Fig. 8K,L). The model simulations, therefore, suggest that TTX-induced upregulation of the h- and T-currents promotes bursting responses in the neurons of hippocampal cell cultures. This finding is in good agreement with the electrophysiological observations under pharmacological manipulations of the intrinsic h- and T-currents (Fig. 7).

## Discussion

By performing a comprehensive electrophysiological study, computational modeling and molecular analysis, we found that hyperpolarization-activated cyclic nucleotide-gated (HCN) and T-type calcium (Ca_V_3) channels play important role in the homeostatic plasticity of primary dissociated neurons as well as hippocampal organotypic slice cultures induced by chronic activity-deprivation.

Previous investigations showed that these channels regulate synaptic integration and intrinsic excitability under normal conditions and contribute to dysfunctional network activity in pathological conditions, especially in epilepsy^26–29^. In the present work we provide evidence that h- and T-currents are upregulated during homeostatic adaptations following 48 h of TTX treatment. Our data show that all subunits of the HCN and T-type calcium channels are present in primary dissociated hippocampal cultures. Blocking spike-mediated transmission increased Ca_V_3.1 mRNA expression, but we did not detect a change in the quantity or plasma membrane localization of the subunit protein. It should be noted that changes in the mRNA levels cannot be interpreted as direct correlates of functional protein expression^30^. Nevertheless, a feasible explanation is that spatial redistribution and/or post-translational modification of T-type Ca channels were induced in a homeostatic manner, leading to increased T-type channel conductances and the establishment of a larger rebound potential, a characteristic physiological signature of T-type Ca-currents.

The action of HCN channels on intrinsic excitability is somewhat bidirectional: on the one hand, HCN channels reduce excitatory postsynaptic potential (EPSP) summation through shunting of the dendritic postsynaptic currents^31,32^. On the other hand, they contribute to the depolarization of the resting membrane (RMP) potential^10^. Additionally, Dyhrfjeld-Johnsen et al.^33^ showed that during fever-induced seizures, upregulated I_h_ evokes dendritic hyperexcitability, which can promote epilepsy. In agreement, activity-deprivation in our cultured neurons induced depolarization of voltage sag positive neurons. Interestingly, we failed to detect TTX-induced changes in HCN mRNA or protein levels in culture lysates, despite of the higher number of cells possessing voltage sag. Notably, we also detected a significant increase in TRIP8b (1a-4) isoform levels, which regulates surface expression of HCN channels in the hippocampus^19,20^. It is known that HCN channel localization primarily determines the extent of voltage sag responses measured in the soma. In agreement, we detected a more proximal HCN1 channel redistribution in CA1 neurons within organotypic hippocampal slices following 48 h of activity-deprivation. Thus, our results show a specific redistribution of HCN channels that is considered as a very effective way of homeostatic upregulation of intrinsic excitability.

It is known that network activity gradually develops in dissociated hippocampal cultures and from DIV12, neurons exhibit robust firing and mature integrative properties^34^. Our data show that two days of TTX treatment started between DIV12 to 14 increased burst activity. This is in agreement with earlier observations on treating immature cultures with TTX for 9 days, starting from DIV5 until DIV14^35^. Additionally, our pharmacological experiments showed that HCN and T-type Ca channels participate in the formation of global network bursting activity amplified by TTX-evoked homeostatic regulation. In accordance with previous reports showing that these channels have fundamental role in bursting^36–39^, we found that selective blocking of these channels decreased burst frequency. At the same time, we found no changes in other burst parameters or the mean firing rate. It is known that T-type voltage-gated channels and HCN channels work together and regulate the firing frequency during post-inhibitory rebound, first spike latency and spike precision in the deep cerebellar nuclear neurons^40^. In addition, HCN1 channels were reported to create signaling complexes with certain Ca_V_3 subunits^41^. Nevertheless, we cannot rule out the possibility that sodium channels are also affected by activity-deprivation as this has been verified by earlier reports using similar model systems^11^. It is likely that differential regulation of sodium, HCN and T-type calcium channels all contribute to the formation of the bursting phenotype during homeostatic plasticity.

Homeostatic regulation of intrinsic excitability has been described as an efficient way to prevent neurons from becoming hyper- or hypoactive in case of sustained loss of neuronal inputs or highly elevated neuronal activity, respectively. Initial research on activity-deprived neurons identified several voltage-dependent membrane conductances, including mostly potassium currents^42^, which are associated with spike generation and are subjects of homeostatic regulation. Indeed, up- or downregulation of such conductances can have a profound effect on the excitability of neurons often manifesting as changes in the rheobase or input–output gain parameters. Our previous experimental data on extended amygdala neurons^43^, as well as present computational models, have shown that homeostatic regulation of other voltage-gated currents including T-type and HCN channels can dramatically alter the neuron’s integrative properties while standard parameters of their static excitability appear to be less influenced. Considering these, even those intrinsic changes that introduce minor shifts in the static input–output functions of neurons (e.g. LTP of intrinsic excitability) can have a robust functional impact when neurons operate under fluctuating synaptic inputs. Our present model simulations also support this idea by showing that upregulation of both the h- and T-currents boosts firing under the action of excitatory synaptic inputs more than firing under standard current step stimulation.

In summary, our data show that in hippocampal neurons, HCN and T-type voltage-gated calcium channels participate in the activity-dependent homeostatic regulation of their integrative properties facilitating synchronization of network activity and bursting.

## Materials and methods

### Ethical approval

All experiments have been approved by the institutional Ethical Committee on Animal Experimentation as well as by the National Scientific Ethical Committee on Animal Experimentation (authorized as #PEI/001/1108-4/2013).

### Animal handling

CD1 mice were housed in the local animal facility at 22 ± 1 °C with 12-h light/dark cycles and accessing food and water ad libitum. Animals were maintained and handled in accordance with the Guidelines for Accommodation and Care of Animals, according to the European Convention for the Protection of Vertebrate Animals Used for Experimental and Other Scientific Purposes.

### Cell culture

Primary cultures of embryonic hippocampal neurons were prepared from CD1 mice on embryonic day 17–18. Pregnant animals were sacrificed by cervical dislocation. Embryonic hippocampi were isolated aseptically and hippocampal tissue was digested and triturated according to Czöndör et al.^44^. Cells were seeded onto poly-l-lysine-laminin (Sigma-Aldrich) -coated glass coverslips in 24-well plates at 6.3–6.8 × 10^4^ or 6-well plates at 4.7–5 × 10^4^ cells/cm^2^ density and cultivated at 37 °C in 5% CO_2_ atmosphere. Neurobasal medium (Invitrogen) containing 2% B27 supplement (Invitrogen), 0.5 mM Glutamax (Gibco) and 5% FCS (Invitrogen) was used for plating and for a complete medium change on the first day after plating (DIV1). On the 5th, 9th and 12th day after plating, third of the culture medium was changed to FCS-free fresh medium. To inhibit glial cell division, 10 µM cytosine β-d-arabinofuranoside (Sigma-Adrich) was added to the cultures between DIV4 to 6. To achieve a complete blockade of firing, cell cultures were treated with 1 µM tetrodotoxin (TTX, Tocris) between DIV12–14.

### Organotypic slice culture

Organotypic hippocampal slices were prepared from CD1 mice on postnatal day 7–8. Animals were sacrificed by decapitation. Brains were removed and immediately placed into chilled dissection medium (92 mM NaCl, 2.5 mM KCl, 1.25 NaH_2_PO_4_ × 2H_2_O, 18 mM NaHCO_3_, 25 mM HEPES, 25 mM glucose, 5 mM sodium ascorbate, 2 mM thiourea. 3 mM sodium pyruvate, 10 mM MgSO_4_, 0.5 mM CaCl_2_ × 6H_2_O), where cerebellum and olfactory bulb were removed. Forebrains were embedded in 2% agarose (SeaKem) and fixed on the Teflon stage of a vibratome (Leica VT 1000S) for 350 μm coronal or horizontal sectioning. Hippocampus slices were isolated in chilled dissection medium under the stereomicroscope. A snipped 1000 ml pipette tip was used to gently transfer the hippocampus to a 6-well PTFE membrane insert with 0.4 μm pore size (Millipore). Slices were kept in slice culture medium containing 50% Basal Medium Eagle (Sigma), 25% Hanks Balanced salt Solution (Sigma), 25% heat-inactivated horse serum (Sigma), 18 g/ml glucose solution (Sigma), 0.146 mg/ml l-glutamine (Sigma) and 0,5% Penicillin–Streptomycin mix (Gibco), at 37 °C and in 5% CO_2_ atmosphere. Medium was replaced completely on DIV1 and later on every third day. Organotypic hippocampal slice cultures were treated with 1 µM tetrodotoxin (Tocris) between DIV10-15 for a duration of 48 h in a way that one hemisphere of a given slice was kept as control while the other was treated with TTX.

### Electrophysiology

#### Patch clamp recordings in dissociated cell cultures

Electrophysiological recordings were performed under an Axiovert 200 microscope (Zeiss). Spontaneous activity and evoked responses were recorded at room temperature (21–23 °C) in whole-cell conditions using a MultiClamp 700B amplifier (Molecular Devices). Intracellular voltage and current traces were sampled at 20 kHz and stimulus command waveforms were generated by the data acquisition software DASYLab v.11 (DASYTec USA, National Instruments; https://www.mccdaq.com/dasylab-index). Patch pipettes (7–10 MOhm) were pulled from standard wall glass of 1.5 mm OD (Warner Instruments). The composition of the bath solution (ACSF) was (in mM): NaCl 140, KCl 5, CaCl_2_ 2, MgCl_2_ 1, HEPES 5, d-glucose 10; pH set to 7.45, while patch electrodes were filled with the following solution (in mM): K-gluconate 100, KCl 10, KOH 20, MgCl_2_ 2, NaCl 2, HEPES 10, EGTA 0.2, D-glucose 5; pH set to 7.3. Miniature excitatory postsynaptic currents (mEPSCs) were acquired at − 60 mV holding potential in the presence of 500 nM tetrodotoxin (Tocris). To record voltage responses under current step stimulation (stepwise current commands of 350 ms duration, starting at − 200 pA and incremented by + 5 pA), neurons were synaptically isolated from network inputs using a combination of CNQX (10 µM), AP5 (40 µM) and bicuculline (30 µM; all from Tocris). Analysis of mEPSCs, spontaneous firing/bursting patterns as well as the evoked responses was performed using software developed by A. Szücs (NeuroExpress). Bursts were defined as clusters containing at least 3 action potentials with adjacent spikes occurring within 0.8 s. We note that in control cultures regular bursting was not always observed and for such preparations the parameter referred to as burst cycle period (BCP) was not obtained. In current step experiments, multiple physiological parameters including the resting membrane potential, rheobase, input resistance and relative voltage sag were determined for each cell.

50 µM ZD7288 (Tocris) and 500 µM NiCl_2_ (Sigma-Adrich) were used to selectively block HCN and T-type calcium channels, respectively.

#### Patch clamp recordings in organotypic slice cultures

Electrophysiological recordings were performed under a Nikon Eclipse FM microscope. During the experiments, slices were perfused with 37 °C, 95% O_2_ plus 5% CO_2_-saturated ACSF containing blockers of synaptic transmission as performed with the dissociated cultured neurons (see above). The experimental equipment was the same as used with the cell cultures. To visualize the patched cells, 1 mM biocytin (Tocris) were added to the patch pipette solution. Voltage responses under current step stimulation were recorded and their analysis was performed as described under cell culture preparations.

### Computational modeling

To simulate the effects of h-current and T-current manipulation, first we implemented a computational model neuron consisting of a dendritic, a somatic and an axonic compartment (passive membrane properties are listed in Supplementary Table S1; see also Hernath et al.^45^ All intrinsic voltage-dependent currents were calculated as

$$I_{i} = g_{i} m_{i}^{p} h_{i} \left( {E_{i} - V} \right) ,$$

where *i* represents the individual current type, *g*_*i*_ is the maximal conductance of the current, *m*_*i*_ is the activation variable, *p* is the exponent of the activation term, *h*_*i*_ is the inactivation variable (either first-order or absent) and *E*_*i*_ is the reversal potential. Differential equations for the activation (*m*) and inactivation (*h*) shared the same form (*x* being either *m* or *h*):

$$\frac{dx}{{dt}} = \frac{{x_{\infty } \left( V \right) - x}}{{\tau_{x} \left( V \right)}} ,$$

where voltage-dependent steady-state activation and inactivation were described by sigmoids:

$$x_{\infty } \left( V \right) = \frac{1}{2} + \frac{1}{2}{\text{tanh}}\left( {\frac{{V - V_{x,1/2} }}{{V_{x,sl} }}} \right) .$$

The midpoint *V*_*x,*1/2_ and slope *V*_*x,sl*_ parameters of the sigmoids and the other kinetic parameters are shown in Supplementary Table S2. Time constant of the activation and inactivation were bell-shaped functions of the membrane potential:

$$\tau_{x} \left( V \right) = \left( {\tau_{x,max} - \tau_{x,min} } \right)\left[ {1 - \tanh \left( {\frac{{V - V_{\tau x,1/2} }}{{V_{\tau x,sl} }}} \right)^{2} } \right] + \tau_{x,min} .$$

The Ca-dependent K-current and internal Ca-dynamics were based on the formalism in Canavier and Landry^46^. Synaptic currents were described using a first-order kinetics of transmitter release^47^ as:

$$I_{syn} = g_{syn} S\left( {E_{syn} - V} \right),$$

where *S* is the instantaneous synaptic activation term yielding the following differential equation:

$$\frac{dS}{{dt}} = \frac{{S_{\infty } \left( {V_{pre} } \right) - S}}{{\tau_{syn} \left( {1 - S_{\infty } \left( {V_{pre} } \right)} \right)}} .$$

The steady-date synaptic activation term depends on the presynaptic membrane potential as

$$S_{\infty } \left( {V_{pre} } \right) = \tanh \left( {\frac{{V_{pre} - V_{th} }}{{V_{slope} }}} \right) ,$$

when *V*_*pre*_ > *V*_*th*_, otherwise $S_{\infty } \left( {V_{pre} } \right) = 0$. *V*_*pre*_ denotes the presynaptic membrane potential waveform that is stored in ASCII files and designed prior to the model runs^48^. The reversal potential of the excitatory and inhibitory synaptic connections was 0 and − 72 mV, respectively. Other synaptic parameters were as follows: *τ*_*syn*_ = 10 ms; *V*_*th*_ =  − 40 mV; *V*_*slope*_ = 20 mV.

### Quantitative real-time PCR

Total RNA was isolated from DIV14-16 old cultures with Nucleospin RNA kit (Macherey–Nagel) and cDNA was synthetized by the Maxima First Strand cDNA Synthesis Kit (Thermo Fisher Scientific). Quantitative real-time PCR (RT-qPCR) was performed with CFX96 Real-Time Sytem (BioRad) using Maxima SYBR Green qPCR master mix (Thermo Scientific). The amplification program was as follows: step 1: 95 °C for 10 min, step 2: 95 °C for 15 s, step 3: 55 °C for 30 s, step 4: 72 °C for 30 s, step 5: repeat step 2–4 for 39 cycles, step 6: 72 °C for 10 min, step 7: run melt curve analysis 65 °C to 95 °C increment with 0.5 °C for 5 s. GAPDH expression level was used to determine the relative expression of the target genes, CACNA1G (Ca_V_3.1, NM_001177890.1), CACNA1H (Ca_V_3.2, NM_001163691.1), CACNA1I (Ca_V_3.3, NM_001044308), HCN1 (NM_010408.3), HCN2 (NM_008226.2), HCN3 (NM_008227.1), HCN4 (NM_001081192.1), TRIP8b (NM_021483.5), TRIP8b (1a-x^15^). Supplementary Table S3. summarizes the used primer sequences (Integrated DNA Technology) in our measurements. Data were analyzed by the ΔΔCt method using the CFX Manager software (version 3.1, BioRad, https://www.bio-rad.com/en-hu/sku/1845000-cfx-manager-software?ID=1845000).

### Protein extraction, cell surface biotinylation, western blot

Whole-cell extracts were obtained by solubilizing primary hippocampal neurons in lysis buffer (1% NP-40, 0.02% SDS, 50 mM NaF) containing protease and phosphatase inhibitors (Complete Mini Protease and PhosSTOP Phosphatase Inhibitor Cocktails; Roche) in phosphate-buffered saline (PBS). Lysates were clarified by centrifugation at 13,000×*g* for 10 min at 4 °C.

To determine the ratio of HCN1 and T-type calcium channels surface localization, we performed cell surface biotinylation. Before the lysis, cells were treated at 4 °C for 30 min with 0.5 mg/ml succinimidyl 2-(biotinamido)-ethyl-1,3′-dithiopropionate (EZ-LinkTM Sulfo-NHS-SS-Biotin; Thermo Scientific) in PBSCM (0.1 mM CaCl_2_, 1 mM MgCl_2_ in PBS; pH 8.0), and washed twice for 7 min in 20 mM glycine (Roth) PBSCM to quench the unreacted biotinylation reagent. Culture lysates were prepared in lysis buffer, and 15 μl was directly frozen as the “total” sample. The rest was incubated overnight at 4 °C with 30 µl of NeutrAvidin-coupled agarose beads (NeutrAvidin Agarose Resins; Thermo Scientific). Beads were washed with ice-cold lysis buffer, and biotinylated proteins were eluted with 50 mM reduced l-glutathione for 15 min at 4 °C. Extracted proteins were subjected to SDS–PAGE and blotted onto polyvinylidene difluoride (Millipore) or nitrocellulose (Thermo Fisher Scientific) membrane. After blocking with 0.5% blocking reagent (Roche Diagnostics; Sigma-Aldrich) in Tris buffer containing 0.05% Tween-20 and 0.1% NaN_3_, membranes were probed with specific antibodies as follows: anti-HCN1 (rabbit, 1:1000; ab176304; Abcam), anti-Ca_V_3.1 (rabbit, 1:4000, 152,503; Synaptic Systems), anti-TRIP8b (exon4) (clone 212/3, mouse, 1:1000, 75–208; NeuroMab) and anti–βIII-tubulin (mouse, 1:10,000; 11-264-C100; Exbio).

Signals were visualized with horse radish peroxidase (HRP)-coupled secondary antibodies (1:20,000; #115-035-005 and #111-035-003; Jackson) using the Luminata Crescendo Western HRP substrate (Millipore). Average intensity values were calculated using Image Studio Lite 5.0 (Li-Cor, https://www.licor.com/bio/image-studio-lite/).

Total HCN1 or Ca_V_3.1 signal intensity values were normalized to their corresponding βIII-tubulin levels. Biotinylated Ca_V_3.1 signal intensity values were normalized to the corresponding total Ca_V_3.1 signal from the same sample. All ratios detected within the same blots were normalized to the average of the control values. Normalized values from 4 to 19 independent cultures were averaged.

### Immunohistochemistry and confocal microscopy

Organotypic hippocampal slice cultures were fixed between DIV12–DIV17 in 4% PFA in PBS for 1 h at room temperature and permeabilized with 1% TritonX100 overnight at 4 °C. Slices were blocked for 1 h in 20% BSA, 0.3% TritonX100 at room temperature, followed by incubating for 2 days with anti-HCN1 antibody (mouse, clone70/28; #MABN20, Millipore) in 2% BSA, 0.5% TritonX100 at 4 °C. After thorough wash with PBS, slices were incubated for 3 h with anti-mouse-AlexaFluor647 (goat F(ab’)2 fragment, #A21237, Life Technologies) in 2% BSA, 0.5% TritonX100, at room temperature. Biocytin-filled recorded cells were visualized with streptavidin-TRITC (#016-020-084, Jackson ImmunoResearch). Cultures were mounted in ProLong Diamond with DAPI (Thermo Scientific).

Images were taken with an upright Zeiss LSM 800 confocal microscope, using a Plan-Apochromat × 20/0.8 (Zeiss) objective. Images were captured as z-stack tiles. To define HCN1 channels location within the CA1 region, average intensity profile was determined along a 100 μm wide line fitted perpendicularly from the str. pyramidale to the str. lacunosum molecular using the ZEN software (version 2.6, Zeiss, https://www.zeiss.com/microscopy/int/products/microscope-software/zen.html). DAPI staining was used to determine the location of stratum pyramidale. In each slice, intensity values were normalized to the average HCN1 intensity within the str. pyramidale. During image acquisition, similar microscopy settings were used and all images were processed uniformly.

### Statistical analyses of the data

Student’s t test or non-parametric Mann–Whitney tests were used for statistical evaluation unless otherwise indicated. SPSS Statistics (version 25, IBM, https://www.ibm.com/analytics/spss-statistics-software) was used to calculate statistics. Data are displayed as mean ± SEM, unless otherwise indicated. p values were accepted statistically significant as *p < 0.05, **p < 0.01, ***p < 0.001.

## Supplementary Information

Supplementary Information.
