# Supplementary material for: Homeostatic plasticity and burst activity are mediated by hyperpolarization-activated cation currents and T-type calcium channels in neuronal cultures
Source: Sci Rep. 2021 Feb 5;11:3236. doi: 10.1038/s41598-021-82775-3 (PMC7864958; doi:10.1038/s41598-021-82775-3)
Supplement: Supplementary file 1 — Supplementary Information. [file 41598_2021_82775_MOESM1_ESM.pdf]

Homeostatic plasticity and burst activity are mediated by hyperpolarization-activated cation currents and T-type calcium channels in neuronal cultures

Anikó Rátkai[1], Krisztián Tárnok[1], Hajar El Aouad[1], Brigitta Micska[1], Katalin Schlett[1,*] and Attila Szücs[1,*]

[1]Department of Physiology and Neurobiology, Institute of Biology, Eötvös Loránd University, Budapest, Hungary

*corresponding authors: Katalin Schlett: schlett.katalin@ttk.elte.hu

Attila Szücs: aszucs@caesar.elte.hu

Supplementary Figure

Supplementary Figure S1.

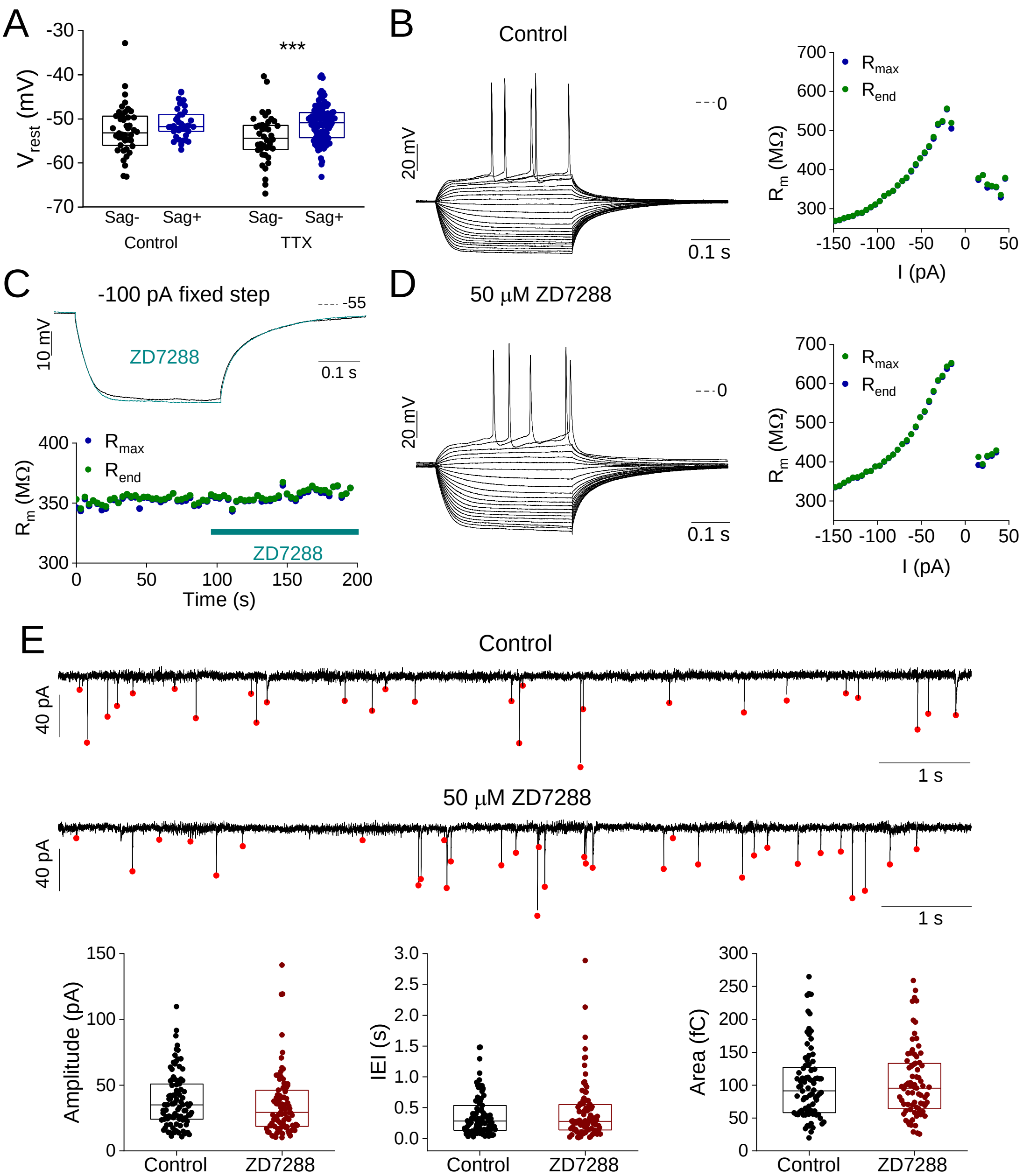

Supplementary Figure S1. (A) Resting membrane potential data recorded in control and TTX-treated dissociated cultures and grouped according to the presence of voltage sag. TTX-treatment evoked significantly depolarized resting membrane potential in sag+ cells. Representative figures show a voltage sag-lacking neuron during current step protocol in control **(B)** and 50 µM ZD7288-treated **(D)** condition. The input/output curves are also presented on the right. **(C)** The top picture shows a representative trace using a fixed -100 pA

current step before (black) and after (turquoise) the application of 50 μM ZD7288. The bottom graph shows the temporal input resistance changes during the whole fixed step protocol. **(E)** Representative 10 sec-long voltage clamp recordings of miniature excitatory postsynaptic currents (mEPSCs) from the same neuron, before (control) and during the 50 μM ZD7288 treatment, respectively. Quantification at the bottom reveals that ZD7288 did not cause any significant changes in the amplitude (left), the inter-event intervallum (IEI; middle) or the area (right) of mEPSCs .

Supplementary Figure S2

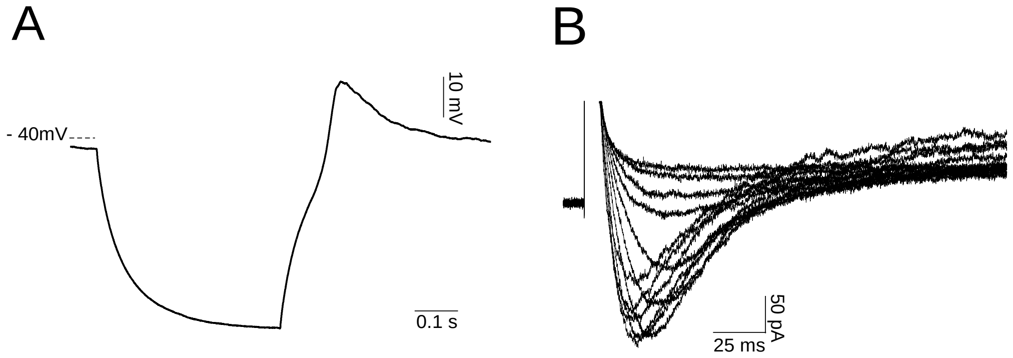

Supplementary Figure S2. A: T-current mediated post-inhibitory rebound (PIR) potential measured in response to a negative current step (-100 pA) in the presence of ZD7288. Note that this cell exhibits no voltage sag, but still a robust PIR potential is generated. **B:** T-current activation profile from the same cell (n=12 repeats). The measuring ACSF contained: 10 µM CNQX, 40 µM AP-5, 30 µM bicuculline, 50 µM ZD7288, 0.5 µM TTX and 500 µM 4-AP.

Supplementary Figure S3.

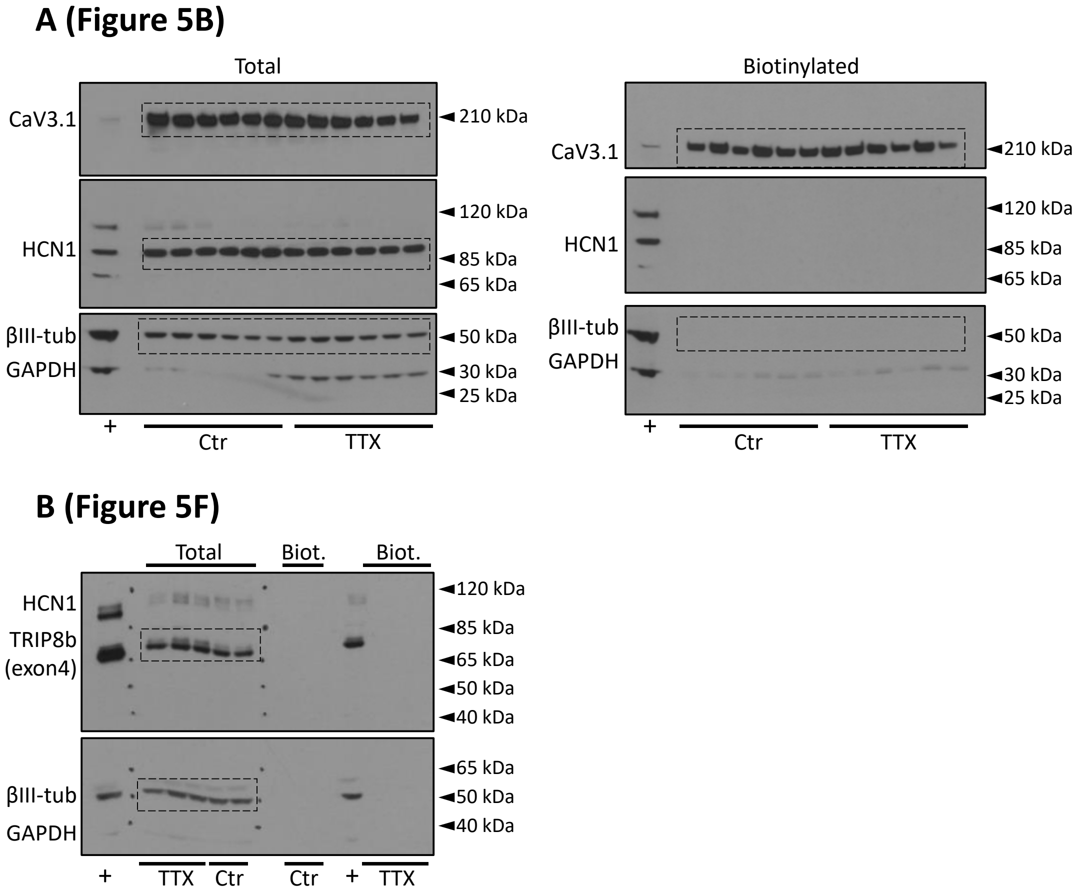

Supplementary Figure S3. Complete images of western blot results. Dashed rectangles show the cropped ROI from the corresponding blots. Adult mouse telencephalon lysates served as positive controls (+). Molecular weight markers are indicated on the right side of the blots. Dotted line and ✂ show the cutline of the membranes.

Supplementary Tables

Supplementary Table 1.

Soma		Axon		Dendrite		Coupling		
g_{leak} [nS]	C_m [pF]	g_{leak} [nS]	C_m [pF]	g_{leak} [nS]	C_m [pF]	g_{sx} [nS]	g_{sd} [nS]	E_{leak} [mV]
1.5	70	1.5	20	1.0	10	18	16	-58.0

Supplementary Table S1. Passive membrane parameters of the hippocampal neuron model. g_{sx} is the electrical coupling conductance between the soma and axon compartments. g_{sd} indicates the coupling between the soma and dendrite.

Supplementary Table 2.

Current	g [nS]	E [mV]	S [%]	A [%]	D [%]	p	$V_{m,1/2}$ [mV]	$V_{m,sl}$ [mV]	$V_{h,1/2}$ [mV]
Na	8000	55	25	75	0	3	-24	14	-50
Na_P	1.0	50	100	0	0	1	-26	14	
H	3.0	-35	33	0	67	1	-65	-16	
K_d	400	-72	25	75	0	4	-20	15	
M	6.0	-75	50	50	0	1	-28	17	
Ca_T	25	90	100	0	0	2	-47	14	-81

Current	$V_{h,sl}$ [mV]	$\tau_{m,max}$ [ms]	$\tau_{m,min}$ [ms]	$V_{\tau m,1/2}$ [mV]	$V_{\tau m,sl}$ [mV]	$\tau_{h,max}$ [ms]	$\tau_{h,min}$ [ms]	$V_{\tau h, 1/2}$ [mV]	$V_{\tau h, sl}$ [mV]
Na	-14	0.9	0.1	-64	30	8	0.5	-70	30
Na_P		3.0	0.2	-70	50				
H		250	50	-55	30				
K_d		10	0.7	-68	30				
M		120	20.0	-80	100				
Ca_T	-13	5.0	0.8	-66	30	50	5	-73	25

Supplementary Table S2. Parameters of the voltage-dependent currents for the hippocampal neuron model. S, A and D indicate the percentage of conductance allocated for the somatic, axonic and dendritic compartments, respectively.

Supplementary Table 3.

Gene name	Forward sequence (5'-3')	Reverse sequence (5'-3')
CACNA1G	GCGACTAAAGAGGCTGGAGA	GTACACAGGTGGTGGACGAG
CACNA1H	CTCACAGCCAGGAGGGTTAG	TGCTTATCTCCAGCGCGTTA
CACNA1I	CATCTTTGGCATCATCGGCG	TCTCGTCGTCCTCTTCTGGT
GAPDH	TGGTGAAGGTCGGTGTGA	ATGAAGGGGTCGTTGATGGC
HCN1	AGCACCGATACCAAGGCAAG	CATGGCCGTCACGAAATTGG
HCN2	CATGACCTACGACCTGGCAA	TGAAGAGCGCGAACGAGTAG
HCN3	AACAAGTTCTCTCTCCGGGTC	CCCACAGGCAGAACTATGAGG
HCN4	TGGAGACTCGCATTGACTCG	CCAATGAGGTTCACGATGCG
TRIP8b	TCACTGTGGAACCGTCTTGG	GACCGCTTCTCTGTAGGCAC
TRIP8b (1a-x)	GAATGTACCAGGGACACATGCAGGG	TGGATGTCACTGGCTTTGCAATGGC

Supplementary Table S3. Primer sequences used in RT-qPCR experiments.
